# Bacterial extracellular vesicles – brain invaders? A systematic review

**DOI:** 10.3389/fnmol.2023.1227655

**Published:** 2023-09-14

**Authors:** Anna Kaisanlahti, Sonja Salmi, Sohvi Kumpula, Sajeen Bahadur Amatya, Jenni Turunen, Mysore Tejesvi, Nadiya Byts, Terhi Tapiainen, Justus Reunanen

**Affiliations:** ^1^Biocenter Oulu, University of Oulu, Oulu, Finland; ^2^Research Unit of Translational Medicine, University of Oulu, Oulu, Finland; ^3^Disease Networks Research Unit, Faculty of Biochemistry and Molecular Medicine, University of Oulu, Oulu, Finland; ^4^Research Unit of Clinical Medicine, University of Oulu, Oulu, Finland; ^5^Department of Ecology and Genetics, Faculty of Science, University of Oulu, Oulu, Finland; ^6^Department of Pediatrics and Adolescent Medicine, Oulu University Hospital, Oulu, Finland

**Keywords:** microbiota, bacteria, extracellular vesicles, brain, blood–brain barrier

## Abstract

**Introduction:**

Knowledge on the human gut microbiota in health and disease continues to rapidly expand. In recent years, changes in the gut microbiota composition have been reported as a part of the pathology in numerous neurodegenerative diseases. Bacterial extracellular vesicles (EVs) have been suggested as a novel mechanism for the crosstalk between the brain and gut microbiota, physiologically connecting the observed changes in the brain to gut microbiota dysbiosis.

**Methods:**

Publications reporting findings on bacterial EVs passage through the blood–brain barrier were identified in PubMed and Scopus databases.

**Results:**

The literature search yielded 138 non-duplicate publications, from which 113 records were excluded in title and abstract screening step. From 25 publications subjected to full-text screening, 8 were excluded. The resulting 17 publications were considered for the review.

**Discussion:**

Bacterial EVs have been described with capability to cross the blood–brain barrier, but the mechanisms behind the crossing remain largely unknown. Importantly, very little data exists in this context on EVs secreted by the human gut microbiota. This systematic review summarizes the present evidence of bacterial EVs crossing the blood–brain barrier and highlights the importance of future research on gut microbiota-derived EVs in the context of gut-brain communication across the blood–brain barrier.

## Graphical Abstract

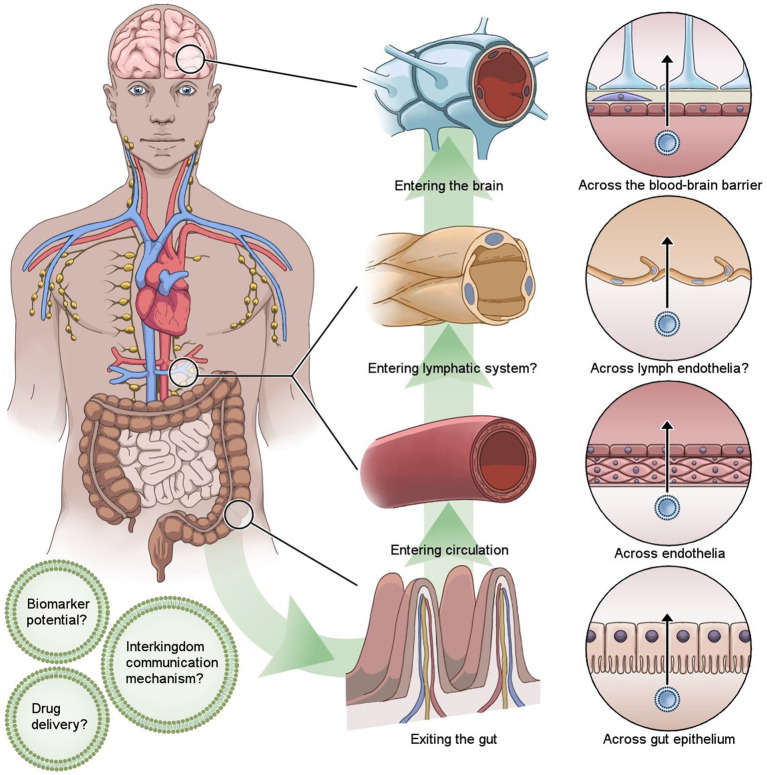

## Introduction

An increasing number of diseases have been associated with changes in the gut microbiota composition. While these changes have been characterized in detail in diseases such as inflammatory bowel disease (Hansen et al., 2012) and metabolic syndrome (Everard et al., 2013), more recent data have suggested alteration of the gut microbiota composition also as a part of pathogenesis of neurodegenerative diseases, such as Parkinson’s disease (Cosma-Grigorov et al., 2020) and Alzheimer’s disease (Vogt et al., 2017). However, it is still unclear how the changes in the gut microbiota relate to the pathological processes observed in the brain.

A few plausible mechanisms for crosstalk between the brain and the gut microbiota have been suggested, including the altered responses of hypothalamus-pituitary axis (Ait-Belgnaoui et al., 2012), immune system (Cheng et al., 2019) and the activation of the vagus nerve (Bravo et al., 2011). Recently, bacterial extracellular vesicles (EVs) secreted by the gut microbiota have been postulated to be one of the key mechanisms contributing to the gut-brain communication (Cuesta et al., 2021). EVs are nano-sized, round, double membrane encapsulated structures that are an essential part of cell biology of both eukaryotes and prokaryotes. EVs are secreted by both gram-negative (Dorward and Garon, 1990) and gram-positive bacteria (Liu et al., 2018), can harbor a variety of biomolecules as their cargo and possess ability to cross biological barriers in the body (Stentz et al., 2018; Sarshar et al., 2022). Blood–brain barrier (BBB) forms a physical and metabolic barrier between the brain and circulation to prevent the entry of harmful chemicals to the brain (Abbott et al., 2006). BBB consists of endothelial cells binding together with tight junctions, the basement membrane, pericytes within the basal lamina that regulate the capillaries and subsequently brain blood flow, and finally astrocytes (Obermeier et al., 2013). While several studies have reported that bacterial EVs can indeed cross BBB, the mechanisms of the passage have remained largely unknown.

In gram-negative bacteria EVs are formed by budding from the outer membrane, which requires detachment of the outer membrane from the peptidoglycan layer. The detachment of the layers requires local changes in the outer membrane in order to maintain its stability, such as breaking the covalent and non-covalent bonds between the constituents of the outer membrane and peptidoglycan layer (Deatherage et al., 2009), repulsion between resulting anionic charges (Beveridge, 1999), and subsequent change in the hydrostatic pressure in the periplasmic space (Schwechheimer et al., 2013) EVs secreted by gram-negative bacteria are also referred to as outer membrane vesicles (OMV) and are considered to be 20–300 nm diameter in size (Avila-Calderón et al., 2021). Another mechanism for EV formation in gram-negative bacteria is through cell death when outer inner membrane vesicles (OIMVs) and explosive outer membrane vesicles (EOMVs) are formed. OIMVs have a cytoplasmic membrane of gram-negative bacteria, a peptidoglycan layer and an outer membrane (Pérez-Cruz et al., 2013), while EOMVs have only an outer membrane (Devos et al., 2017).

The EV formation in gram-positive bacteria has been suggested to take place through membrane budding and cell lysis (Brown et al., 2015). In this process, in order to reach extracellular space, the EVs must pass through the thick peptidoglycan layer. During cell lysis this is enabled by endolysin enzyme (Andreoni et al., 2019), but the exact mechanism for EV formation in membrane budding remains largely unknown. As compared to EVs secreted by gram-negative bacteria, the EVs secreted by gram-positive bacteria have a larger size range of 20–400 nm (Díaz-Garrido et al., 2021). EVs secreted by gram-positive bacteria are also referred to as cytoplasmic membrane vesicles (CMVs), due to a lack of an outer membrane (Toyofuku et al., 2019).

While cargo embedded to bacterial EVs varies by species, the same bacteria can also produce differently loaded EVs depending on the environmental cues (Lynch et al., 2019). The EVs secreted by gram-negative bacteria often harbor molecules originating from the outer membrane, cytoplasmic membrane, peptidoglycan layer and periplasm (Beveridge, 1999), including heat shock proteins, superoxidase dismutases, adhesins, toxins, lipopolysaccharides of the outer membrane (Wispelwey et al., 1989), small RNAs (Han et al., 2019) and other pathogen associated molecular patterns (PAMPs). In addition, bacterial DNA has been observed in EVs secreted by gram-negative bacteria (Pérez-Cruz et al., 2013).

Extracellular vesicles from gram-positive bacteria lack LPS and periplasmic components but carry similar types of cargo molecules as EVs from secreted by gram-negative bacteria, including peptidoglycan, lipids, proteins, and nucleic acids (Brown et al., 2015). EVs secreted by the gram-positive bacteria have been reported to contain lipoteichoic acid, phosphatidylglycerol, cardiolipin (Resch et al., 2016), DNA (Klieve et al., 2005), toxins, enzymes and other proteins, short fatty acids (Olaya-Abril et al., 2014), microRNA, lipids and fluids (Brown et al., 2015). Gram-positive bacteria have been reported to use EVs for gene transfer (Klieve et al., 2005) and CMVs to ship bacteriophage receptors and bacteriophages, thus rendering bacteriophage resistant cells subjective to phage invasion (Toyofuku et al., 2017; Tzipilevich et al., 2017).

In general, bacterial EVs can harbor a variety of neurotransmitters as their cargo, including dopamine, serotonin, noradrenaline and enzymes contributing to synthesis of these molecules. Bacterial EVs can also harbor short chain fatty acids that are able to influence the function of neurons and microglia (Haas-neill and Forsythe, 2020).

*Bacteroidetes*, one of the major bacterial phyla constituting the human gut microbiota, are known to produce enzymes aiding the digestion of nutrients and send them to the gut lumen *via* EV secretion (Elhenawy et al., 2014). These enzymes include hydrolases that degrade complex carbohydrates (Rakoff-Nahoum et al., 2014), inositol polyphosphatases that break down dietary phytate to phosphates, inositol phosphates and inositol (Stentz et al., 2014). The commensal *Bacteroides fragilis* has been reported to secrete EVs containing neurotransmitter gamma-aminobutyric acid (GABA) and its precursors glutamate and α-ketoglutarate, while pathogenic *Bacteroides fragilis* has been reported to secrete EVs embedded with histidine decarboxylase, an enzyme catalyzing histamine synthesis (Zakharzhevskaya et al., 2017).

While bacterial EV cargo can reach the cells of biological barriers through different methods of endocytosis, intact bacterial EVs have been suggested to cross epithelial and endothelial layers using paracellular and transcellular routes. Bacterial EVs passing through the intestinal barrier and entering circulation is supported by findings of gut microbiota-associated EVs in dendritic cells of lamina propria (Shen et al., 2012), urine (Lee et al., 2017), and blood (Chang et al., 2021). Moreover, it has been reported that in mice, bacterial EVs in blood represent the gut microbiome composition of the host (Park et al., 2017). To pass through the gut epithelial layer, EVs can plausibly use transcellular transmigration through caveolae-mediated endocytosis or paracellular transmigration, where EVs pass through the intestinal barrier between the epithelial cells (Jones et al., 2020). However, the routes human gut microbiota EVs take to pass the gut epithelia likely differ between different species and utilize both transcellular and paracellular routes (Stentz et al., 2018).

While there is limited data on human microbiota EVs, EVs produced by pathogens and their translocation across biological barriers have been studied extensively. EVs of pathogenic bacterial strains have been demonstrated to increase the permeability of the intestinal barrier (Turkina et al., 2015) by weakening the extracellular matrix *via* embedded collagenases and hyaluronate lyases (Jeon et al., 2017) or the integrity of physiological barriers by serine protease activity (Hoy et al., 2010; Jarzab et al., 2020). Gingivitis causing bacteria *Aggregatibacter actinomycetemcomitans* and *Porphyromonas gingivalis* have been demonstrated to secrete EVs that increase the expression of vascular endothelial growth factor subsequently altering the permeability of blood vessel endothelium (Suthin et al., 2003). EVs secreted by *Campylobacter jejuni* have been reported to break down E-cadherin of cell junctions and occludin of tight junctions (Elmi et al., 2016). On the other hand, commensal bacteria have been reported to produce EVs that enhance the function of tight junction in the intestinal barrier by increasing zonula occludin expression and subsequently decreasing the paracellular transmigration of EVs of pathogenic strains (Alvarez et al., 2016).

Bacterial EVs have been demonstrated to enter the host cells using different endocytosis mechanisms, including micropinocytosis (Weiner et al., 2016), clathrin-mediated endocytosis (Boisvert and Duncan, 2008), clathrin-independent endocytosis (Norkin et al., 2001; Mondal et al., 2016) and cell membrane fusion (Bomberger et al., 2009). By default, bacterial EVs up taken by the host cells end up in lysosomes for degradation. However, in caveolae-mediated endocytosis EVs are protected by the plasma membrane of the host and are transported to the endoplasmic reticulum or Golgi apparatus, thus allowing bacterial EVs to evade degradation by lysosomes (Lim et al., 2014).

Extracellular vesicles secreted by the gut microbiota can undergo phagocytosis by dendritic cells of the gut lamina propria. For example, the dendritic cells detect EVs secreted by *Bacteroides fragilis* by recognition of capsular polysaccharide A and direct them to phagocytosis *via* TLR2 receptor activation (Shen et al., 2012). This process leads to an increase in the number of regulatory T-cells and transcription of IL-10 and Foxp3, protecting the body from excessive inflammatory response and autoimmunity (Izcue et al., 2009). In addition, EVs secreted by *Lactobacillus rhamnosus* were reported to induce IL-10 and heme oxygenase-1 expression levels in dendritic cells and subsequently increase the number of regulatory T-cells in Peyer’s patches and mesenteric lymph nodes in mice (Al-Nedawi et al., 2015).

In this systematic review we summarize the present evidence of bacterial EVs crossing the BBB and highlight the importance of future research on gut microbiota-derived EVs in the context of gut-brain communication across the BBB.

## Methods

The literature search was performed using PubMed and Scopus databases (query search on 04-08-2023). Literature search parameters were set to find publications that included in their title or abstract one word of each category of the following with options for different spelling: (1) bacteria, microbiota, microbiome (2) extracellular vesicles, outer membrane vesicles (3) brain, blood–brain barrier. Search were conducted in PubMed using the following query: ((“Blood–Brain Barrier”[Mesh] OR “blood brain barrier”[Text Word] OR “Brain”[Mesh] OR Brain [Text Word]) AND (“Extracellular Vesicles”[Mesh] OR “outer membrane vesicle*”[Text Word] OR “extracellular vesicle*”[Text Word])) AND (“Bacteria”[Mesh] OR “Microbiota”[Mesh] OR “bacter*”[Text Word] OR “microbiota”[Text Word] OR “microbiome”[Text Word]). Search were conducted in Scopus using the following query: (TITLE-ABS-KEY(“*bacter*” OR “*microbiota*” OR “*microbiome*”) AND TITLE-ABS-KEY(“*extracellular vesicle*” OR “*outer membrane vesicle*”) AND TITLE-ABS-KEY(“blood–brain barrier” OR “Brain”)). The publications resulting from the literature search were imported to Covidence program for the systematic review and duplicate records were removed (Covidence systematic review software, Veritas Health Innovation, Melbourne, Australia, n.d. Available at www.covidence.org). The abstracts of the publications were screened and records irrelevant to the topic or of other publication type than original research were excluded. Full text screening was performed with the following inclusion criteria: bacterial origin of EVs and incorporation of either *in vivo* or *in vitro* model of EV biodistribution to the brain in the study setting.

## Results

**Figure 1:**
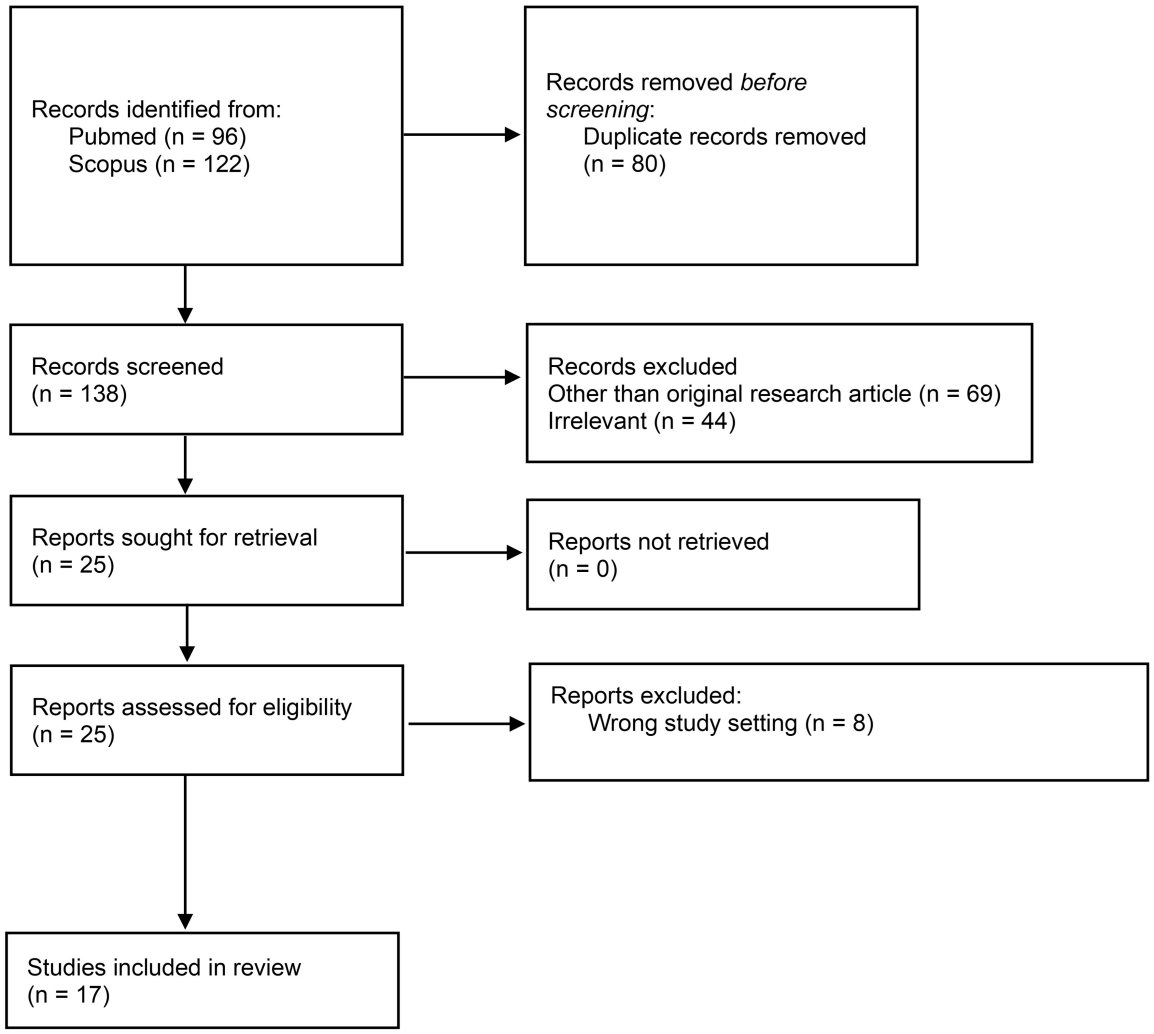
Preferred reporting items for systematic reviews and meta-analyses (PRISMA) flow diagram of the systematic review process.

The literature search yielded 218 publications from which 80 duplicate records were removed. After screening the abstract and title of 138 publications, 113 records were excluded. After full text screening, 8 publications were excluded. As a result, 17 publications were included in this systematic review. PRISMA flow diagram (Page et al., 2021) of the systematic review process is presented in the Figure 1 and the studies included in the review in the Table 1.

**Table 1:** The studies included in the systematic review of bacteria extracellular vesicles crossing through the blood–brain barrier.

EV source	Results reported	Context	Reference
*Porphyromonas gingivalis*	*In vivo*, biodistribution (mouse)	Alzheimer’s disease Periodontitis	Ma et al. (2023)
*Helicobacter Pylori*	*In vivo*, biodistribution (mouse)	*H. pylori* infection-associated neurodegenerative disorders	Palacios et al. (2023)
Escerichia coli	*In vivo*, biodistribution (mouse)	Ischemic stroke	Pan et al. (2023)
*Helicobacter Pylori*	*In vivo*, biodistribution (mouse)	Alzheimer’s disease	Xie et al. (2023)
*Lactobacillus plantarum*, *Bacillus subtilis*, *Akkermansia muciniphila*	*In vivo*, effect on behavior, effect on gene expression (mouse)	Stress	Choi et al. (2022)
*Porphyromonas gingivalis*	*In vivo*, biodistribution (mouse)	Alzheimer’s disease	Gong et al. (2022)
*Salmonella*	*In vivo*, biodistribution (mouse)	Chemotherapy	Mi et al. (2022)
*Echerichia coli*	*In vivo*, biodistribution (mouse)	Translocation of gut microbiota EVs to host cells	Bittel et al. (2021)
*Aggregatibacter actinomycetemcomitans*	*In vivo*, biodistribution (mouse)	Periodontitis	Ha et al. (2020)
*Paenalcaligenes hominis*	*In vivo*, biodistribution (mouse)	Alzheimer’s disease	Lee et al. (2020)
*Akkermansia muciniphila*	*In vivo*, effect on gene expression (mouse)	Serotonin signaling	Yaghoubfar et al. (2020)
*Lactobacillus plantarum*	*In vivo*, effect on behavior, effect on gene expression (mouse)	Stress	Choi et al. (2019)
*Aggregatibacter actinomycetemcomitans*	*In vivo*, biodistribution (mouse)	Periodontitis	Han et al. (2019)
Feces	*In vivo*, biodistribution (mouse)	Alzheimer’s disease	Wei et al. (2020)
*Haemophilus influenzae*	*In vivo*, BBB model (mouse)	Meningitis	Wispelwey et al. (1989)
*Porphyromonas gingivalis*	*In vitro*, BBB model (human)	Alzheimer’s disease Periodontitis	Nonaka et al. (2022)
*Porphyromonas gingivalis*	*In vitro*, BBB model (human)	Periodontitis	Pritchard et al. (2022)

### The evidence of bacterial EVs passing through the blood–brain barrier

The studies providing evidence on bacterial EV passage through BBB highlights an established connection between oral health and neurodegenerative disorders (Han et al., 2019; Ha et al., 2020; Gong et al., 2022; Nonaka et al., 2022; Pritchard et al., 2022; Ma et al., 2023). Indeed, the bacterial EV passage through BBB has been most often studied with oral pathogens, such as *Porphyromonas gingivalis* (Gong et al., 2022; Nonaka et al., 2022; Pritchard et al., 2022; Ma et al., 2023) and *Aggregatibacter actinomycetemcomitans* (Han et al., 2019; Ha et al., 2020). The scope of bacterial EV research regarding BBB crossing also includes diseases involving brain inflammation (Wispelwey et al., 1989; Lee et al., 2020; Palacios et al., 2023; Xie et al., 2023), ischemic stroke (Pan et al., 2023), stress (Choi et al., 2019, 2022), serotonin signaling (Yaghoubfar et al., 2020) and chemotherapy (Mi et al., 2022). A study by Bittel et al. (2021) modelled the translocation of gut microbiota EVs to host distal organs and host cells using *Escherichia coli* (Bittel et al., 2021). In contrast to the several studies mentioned above that examined BBB crossing by single strains of bacteria, a study by Wei et al. (2020) examined the biodistribution of feces-derived EVs in patients with Alzheimer’s disease, representing the EV pool secreted by the entirety of the gut microbiota (Wei et al., 2020) and a study by Choi et al. (2022) reported changes in mouse brain in response to treatment with 3 different probiotics. The studies incorporating bacterial EV passage through BBB are summarized in the Table 1.

### Analysis of blood–brain barrier passage

The ability of bacterial EVs to cross the BBB has been studied *in vivo* using different murine models (Wispelwey et al., 1989; Choi et al., 2019, 2022; Han et al., 2019; Ha et al., 2020; Lee et al., 2020; Wei et al., 2020; Yaghoubfar et al., 2020; Bittel et al., 2021; Gong et al., 2022; Mi et al., 2022; Ma et al., 2023; Palacios et al., 2023; Pan et al., 2023; Xie et al., 2023) and conventional *in vitro* human BBB cell models employing brain endothelial cell monolayers (Nonaka et al., 2022; Pritchard et al., 2022). The majority of the *in vivo* studies employ EV labeling and subsequent imaging analysis in mouse models in their study setting. Imaging analysis is often coupled with other experiments quantifying specific effects in the brain or aiming to describe the mechanism of how bacterial EVs cross this strict biological barrier. In studies involving EV labelling and imaging analysis, a lipid dye for membrane staining is most often employed. Notably, Han et al. (2019) used a combination of membrane staining lipid dye and RNA stain embedded in bacterial EVs (Han et al., 2019). In their study, Mi et al. (2022) measured the fluorescence of doxorubicin (DOX) in DOX-loaded *Salmonella typhimurium* derived EVs thus their study did not involve EV staining (Mi et al., 2022). Similarly, Xie et al. (2023) used Cre-recombinase loaded EVs of *Escherichia coli* in *Rosa26.tdtomato* mice in their study setting instead of EV labelling (Xie et al., 2023). Moreover, in the very early study of Wispelwey et al. (1989), authors modelled BBB permeability through the leucocyte count in cerebrospinal fluid as compared to blood in leukopenia-induced mice (Wispelwey et al., 1989). Three studies reported EV translocation to brain in mouse models solely through changes in brain tissue gene expression and/or effect in behavior (Choi et al., 2019, 2022; Yaghoubfar et al., 2020). In the *in vivo* experiments describing bacterial EV passage through BBB, saline is most often used as a negative control. Noteworthy, Xie et al. (2023) used *Helicobacter pylori* culture medium as their negative control (Xie et al., 2023). Quantification, EV labelling methods and negative controls used in *in vivo* studies of bacterial EVs passing through BBB are summarized in the Table 2.

**Table 2:** Quantification, EV labelling methods and negative controls used in *in vivo* studies of bacterial extracellular vesicles passing through the blood–brain barrier.

Quantification	EV labeling	Negative control	Reference
Confocal microscopy	membrane (FITC)	Saline	Ma et al. (2023)
*In-vivo* fluorescence imaging	membrane (DiR)	Control, control +DiR (control not mentioned)	Palacios et al. (2023)
MRI of ischemic area	NA	Saline, PGZ	Pan et al. (2023)
Confocal microscopy	NA (Cre-recombinase loaded)	Culture medium Unloaded EVs	Xie et al. (2023)
Behavioral tests qPCR	NA	Saline	Choi et al. (2022)
Confocal microscopy	Membrane (DiO)	PBS	Gong et al. (2022)
Excitation light imaging	NA (DOX loaded)	DMEM, free DOX free DOX and bacteria	Mi et al. (2022)
Confocal microscopy	NA (*E. coli*^Cre^ as EV source)	E. coli^GFP^	Bittel et al. (2021)
Intravital imaging	Membrane (DiD)	0 h time point imaging	Ha et al. (2020)
Confocal microscopy	Membrane (FITC)	Saline	Lee et al. (2020)
qPCR	NA	PBS	Yaghoubfar et al. (2020)
Behavioral tests. qPCR	NA	Saline	Choi et al. (2019)
2D-lightsheet fluorescence microscopy	Membrane (DiD) RNA (Syto-RNA select)	PBS	Han et al. (2019)
Confocal microscopy	Membrane (PKH26)	Saline	Wei et al. (2020)
Leucocyte count in CSF vs. blood	NA	Saline	Wispelwey et al. (1989)

### Administration of bacterial EVs

In murine models, EVs have been most often administrated by oral gavage (Lee et al., 2020; Yaghoubfar et al., 2020; Gong et al., 2022; Ma et al., 2023) or as an injection to blood circulation (Mi et al., 2022), either by intracardiac injection (Han et al., 2019) or through the tail vein (Ha et al., 2020; Wei et al., 2020; Palacios et al., 2023; Pan et al., 2023). Gong et al. (2022) reported passage of *Porphyromonas gingivalis* EVs through BBB when administrated by oral gavage to mice when 100 μg of EVs (per EV protein) and 3 days of circulation time was used (Gong et al., 2022). Study setting of Lee et al. (2020) used daily EV dosage of 10 μg/kg normalized to EV protein and LPS content for 5 days (Lee et al., 2020). Another study incorporating EV administration through oral gavage by Yaghoubfar et al. (2020) administrated 10 μg of EVs (per EV protein) to mice daily for 4 weeks (Yaghoubfar et al., 2020). Ma et al. (2023) incorporated both oral gavage and gingiva exposure as EV administration routes to their study setting: in the brain biodistribution assay using gingiva exposure, 2 μg of EVs/day (per protein) were administrated to mice for 5 days (Ma et al., 2023).

A study by Palacios et al. (2023) examined translocation of *Helicobacter pylori* EVs to brain in mice *via* injection to blood circulation (Palacios et al., 2023). In their study Palacios et al. tested a range of EV doses from 5 – 100 μg with circulation time of 24 h and 72 h (Palacios et al., 2023). Pan et al. (2023) reported bacterial EV-mediated delivery of pioglitazone to brain in ischemic stroke mouse model through injection to tail vein with 4 days treatment time (Pan et al., 2023). Mi et al. (2022) used normalization of EV loaded with DOX to DOX stain added to EV solution (2 mg of DOX per kg of body weight) and reported their results within 8 h circulation time (Mi et al., 2022). Wei et al. (2020) reported feces-derived EV passage through BBB in 12 h after administration through the tail vein with EV protein content-based dosage of 50 μM per body weight (Wei et al., 2020). A Study by Han et al. (2019) reported crossing of BBB at 24 h after intracardiac injection to mice using an estimated EV amount of 6.75 × 10^11 particles as measured with nanoparticle tracking analysis (Han et al., 2019). A continuation study by Ha et al. (2020) reported EVs crossing the BBB taking place between 8 and 48 h of circulation when administrated through the tail vein and using estimated particle amount of 3 × 10^8 particles (Ha et al., 2020).

A few studies employed either intragastric administration (Xie et al., 2023) or administration to intraperitoneal space (Choi et al., 2019, 2022). Xie et al. (2023) conducted a biodistribution assay to brain with *Helicobacter pylori*-derived EVs administrated by daily intragastric injection to mice, using 20 μg of EVs (per EV protein; 4 × 10^10 particles) and 5 days treatment time (Xie et al., 2023). Two studies by Choi et al. (2019, 2022) describe stress-ameliorating effect of intraperitoneally-administrated EVs of probiotic bacteria in a mouse model (Choi et al., 2019, 2022). Wispelwey et al. (1989) tested *H. influenzae* EVs in a meningitis rat model with EVs administered with intracisternal injection and dosage normalized to 20 ng of LPS content for circulation times between 2 and 8 h. While no change in the BBB permeability was reported in 2 h time point, a maximal increase was reported to take place at 4 h and decrease significantly toward 8 h. In the same study, a series of EV LPS concentrations were tested with 4 h circulation time, concluding that all the tested concentrations starting from 200 pg. significantly increased the BBB permeability, only exception being the highest concentration 4 μg (Wispelwey et al., 1989).

Besides *in vivo* studies, few studies using conventional BBB *in vitro* modelling with human cells exists to date. In their BBB model consisting of human brain microvascular endothelial cells (HBMEC), Pritchard et al. (2022) reported a change in transendothelial electrical resistance (TEER) in response to a treatment with EVs from *Porphyromonas gingivalis* in a range of 0.1 μg/ml – 100 μg/ml (per measured Nanodrop concentration). Moreover, they reported FITC-Dextran permeation to be constant after 24 h of exposure, with correlation between the BBB permeability and EV concentration (Pritchard et al., 2022). Nonaka et al. (2022) tested the BBB permeability in human cerebral endothelial cell model and observed a significant increase in the BBB model permeability at 4 and 6 h time points in transwells treated with 200 μl of EV solution with 150 μg/ml (per EV protein concentration) (Nonaka et al., 2022). Administration, dosage and treatment times of bacterial EVs used in studies of bacterial EVs passing through BBB are summarized in the Table 3.

**Table 3:** Administration, dosage and treatment times of extracellular vesicles used in studies of bacterial extracellular vesicles passing through the blood–brain barrier.

Administration	EV dosage and normalization	Treatment time	Reference
Exposure to gingiva	2 μg/mouse/mouse/day, per EV protein	5 days	Ma et al. (2023)
Injection through tail vein	5, 10, 20, and 100 μg, per EV protein;	24 h, 72 h	Palacios et al. (2023)
Injection through tail vein	1 mg/kg by PGZ content loaded to EVs	4 days	Pan et al. (2023)
Intragastric administration	20 μg, per EV protein, 4 × 10^10 particles	5 days	Xie et al. (2023)
Intraperitoneal injection	2 μg/100 μL/mouse/day	14 days	Choi et al. (2022)
Oral gavage	100 μg per EV protein per mouse	3 days	Gong et al. (2022)
Intravenous injection	2 mg/kg by DOX content loaded to EVs	8 h	Mi et al. (2022)
NA (oral gavage of whole bacteria)	NA	4 days	Bittel et al. (2021)
Injection through tail vein	3 ×10^8 particles per mouse	4–48 h	Ha et al. (2020)
Oral gavage	10 μg/kg/day per EV protein and 32 ng/kg/day per EV LPS	5 days	Lee et al. (2020)
Oral gavage	10 μg/day/mouse per EV protein	4 weeks	Yaghoubfar et al. (2020)
Intraperitoneal injection	0.1, 0.18, 0.27 μg/kg (5, 2, 7 days)	14 days	Choi et al. (2019)
Intracardiac injection	6.75 ×10^11 particles per mouse	4 h/24 h	Han et al. (2019)
Injection through tail vein	50 μM body weight, per EV protein	12 h	Wei et al. (2020)
Intracisternal injection	20 ng LPS and 200 pg. – 4 μg, per EV LPS	2, 4, 6, 8 h	Wispelwey et al. (1989)
Cell culture treatment	200 μl of 150 μg/ml, per EV protein	4 h/6 h	Nonaka et al. (2022)
Cell culture treatment	0.1–100 μg/ml, per nanodrop concentration	0.5–72 h	Pritchard et al. (2022)

## Discussion

The studies exploring bacterial EVs passage through BBB in murines so far have been done with varying EV sources, administration locations, dosages, circulation times and most importantly with varying methods of quantification. It is important to note that the technique used in the EV administration has a wide effect on implications of the obtained results. While the passaging of bacterial EVs through BBB can be studied with different administration routes, the number of biological barriers EVs have to pass through increases if the administration takes place, e.g., *via* the oral cavity as compared to direct intravenous administration. Thus, the study settings and administration technique need to be planned accordingly paying attention to detail and taking into account the larger context of the study at hand. While the majority of studies tracking bacterial EV passage to the brain employ staining of the EVs and subsequent imaging analysis, the importance of the use of negative controls is highlighted. Thus, control samples representing diffusion of the dye itself need to be incorporated. It is also of importance whether a loading dye targeting the membrane or the cargo is used, in other words, distinguishing whether the staining represents intact EVs or released cargo as well.

The data on the mechanisms how bacterial EVs cross BBB remain limited. In recent studies, one of the leading hypotheses of how bacterial EVs pass through BBB is that they directly alter its permeability. Indeed, many studies on bacterial EVs and the BBB permeability report a decrease in gene expression of tight junction-related proteins in response to bacterial EV administration (Wei et al., 2020; Gong et al., 2022; Nonaka et al., 2022; Pritchard et al., 2022) while a decrease in the number of tight junctions has been demonstrated to directly increase the permeability of BBB (Hartsock and Nelson, 2008). Wei et al. (2020) reported a decrease in claudin-5 tight junction protein expression in the mouse hippocampus in response to EVs isolated from fecal samples of patients with Alzheimer’s disease (Wei et al., 2020). Gong et al. (2022) observed a reduction of zonula occludens-1 (ZO-1), occludin, claudin-5 mRNA expression and occluding protein expression in the hippocampus in response to oral administration of *Porphyromonas gingivalis* to mice (Gong et al., 2022). Nonaka et al. (2022) reported an induced degradation of ZO-1 and occludin in their human *in vitro* BBB cell model (Nonaka et al., 2022). Interestingly, in their study Xie et al. (2023) indicated that *Helicobacter pylori* EVs translocate from stomach to brain through transcellular pathways without disrupting the gastrointestinal or blood–brain barriers (Xie et al., 2023).

In addition to changes in BBB permeability, Pan et al. (2023) and Mi et al. (2022) demonstrated that bacterial EVs are able to” hitchhike” through BBB in neutrophils (Mi et al., 2022; Pan et al., 2023). In their study setting, Mi et al. (2022) used a mouse model in which the brain tumors of the mice were colonized by intravenous injections of bioengineered *Salmonella typhimurium*, a strain characterized by an enhanced tumor-homing and a capability of induction of neutrophil recruitment. They observed that EVs isolated from the culture of *Salmonella typhimurium* and loaded with doxorubicin (DOX) could cross BBB and reach the colonized brain tumor. While this finding demonstrated the role of EVs as carriers mediating the crossing of BBB, it implied that bacterial EVs could not cross BBB in an absence of tumor colonization and subsequent neutrophil infiltration to the brain (Mi et al., 2022). Pan et al. (2023) reported successful delivery of pioglitazone over BBB with *Escherichia coli* EVs in their murine stroke model (Pan et al., 2023). In their study, Pan et al. (2023) assessed pioglitazone-EV uptake to neutrophils, their stability during the hitchhike and BBB penetration capability of neutrophil-engulfed pioglitazone-EVs *in vitro* (Pan et al., 2023).

A number of mechanisms have been suggested for how whole pathogenic bacteria cross BBB in pathologies involving infection of the brain, such as bacterial meningitis (Kim et al., 2005, 2015). More recently, it has been speculated that EVs secreted by these pathogens could be a main factor driving the changes in the BBB permeability to enable infiltration of whole cell bacteria through the barrier (Nonaka et al., 2022). As a demonstration, a study by Kim et al. (2005) reported that *Escherichia coli* is able to pass through BBB in a human *in vitro* cell model through cytotoxic necrotizing factor 1 (CNF1) action (Kim et al., 2005). Later on, it was demonstrated that CNF1 secretion from bacteria cytoplasm is mediated *via* EVs (Davis et al., 2006; Kouokam et al., 2006) and that a deficiency of the protein mediating CNF1 packaging to EVs decreased the ability of the bacteria to invade the microvascular endothelial cells in the human brain (Yu and Kim, 2012). Thus, in addition to the fact that bacterial EVs are able to cross BBB themselves, their contribution to the whole bacterial cell infiltration into the brain needs further evaluation. In addition, the possible presence and role of bacterial EVs needs to be assessed in studies that report a bacterial presence in the brain based on quantification of bacterial biomolecules in brain samples. Thus, it needs to be evaluated if the reported findings are due to translocation of bacterial EVs and their cargo instead of actual presence of whole cell bacteria.

While the effect of EVs derived from oral pathogens in the brain have been extensively studied, little is known about the communication of the brain and the microbiota through EVs in health. The relevance of EV secretion from different human microbiotas, most interestingly gut microbiota, needs to be further evaluated: bacterial EVs are likely to be one of the key aspects of the communication between the vital gut microbiota and the host, so their role in health requires more research. Notably, while it is known that bacterial EVs can modify host immune responses, it is of importance to establish the role of microbiota-derived EVs in the immunomodulation processes in health and disease inside and outside of the scope of neuroinflammation.

While circulation is often considered to be the most relevant route for bacterial EVs’ to enter the brain in regard to their role in disease and their potential use in medical applications, there are few other possible routes for bacterial EVs to reach the brain. There is very little data on the bacterial EV passage through other systems and their relevance especially in a healthy state. Currently, to our best knowledge, there is no data on bacterial EV transport to the brain *via* the lymphatic system and only few studies explore the possibility of bacterial EV trafficking *via* the vagus nerve (Lee et al., 2020) or their effect on the conductivity of the afferent fibers (Al-Nedawi et al., 2015).

Bacterial EVs possess enormous potential in multiple biomedical applications, including vaccine platforms, biomarker discovery, drug delivery and discovery of novel molecules with pharmacological value. Bacterial EVs of gram-negative bacteria are already used in vaccines, although the toxicity of their LPS content is redeemed problematic (Acevedo et al., 2014). EVs of gram-positive bacteria have been suggested for vaccine development due to the absence of LPS in them (Choi et al., 2015). Bacterial EVs have potential to serve as biomarkers in different pathologies that involve invasion by pathogenic bacteria. In addition, the gut microbiota-derived EVs might be used as a marker of gut microbiota dysbiosis associated with several diseases (Hansen et al., 2012; Everard et al., 2013; Vogt et al., 2017; Cosma-Grigorov et al., 2020). Based on their studies in mice, Park et al. (2017) suggested analysis of blood sample-associated bacterial EVs as a technique for screening the dysbiosis of the gut microbiota in patients with neurodegenerative diseases (Park et al., 2017). In addition to blood sampling, changes in the gut microbiota have been screened by analyzing bacterial EVs in urine samples from individuals with autism spectrum disorder (Lee et al., 2017).

The ability to cross biological barriers, to carry variety of biomolecules, and to efficiently protect their combined with high potential for bioengineering render bacterial EVs with enormous potential in drug delivery, including targeted delivery of antibiotics and cytostatic chemotherapy. In their study suggesting bacterial EV hitchhiking across BBB in host immune cells, Mi et al. (2022) demonstrated that doxorubicin could be efficiently target-delivered to *Salmonella typhimurium* strain-colonized glioma with bioengineered EVs from the same strain when EVs were administrated intravenously to mice (Mi et al., 2022). In turn, Pan et al. (2023) demonstrated delivery of pioglitazone to the brain of ischemic mice *via* bacterial EVs (Pan et al., 2023).

In conclusion, bacterial EVs have been characterized to cross BBB in animal models and *in vivo* cell cultures, but the occurrence of this in the context of the human gut microbiota-brain communication remains poorly characterized. Bacterial EVs and their passage through BBB creates possibilities for their use in biomedical applications. Indeed, bacterial EVs can be considered a ready-made, co-evolution driven interkingdom transporting system.

## Data availability statement

The original contributions presented in the study are included in the article/supplementary material, further inquiries can be directed to the corresponding author.

## Author contributions

AK and JR: conceptualization. AK, SS, SA, and JR: methodology. AK: literature review. AK and SK: writing – original draft preparation. AK, SS, SA, JT, MT, NB, TT, and JR: writing – review and editing. SS: graphical abstract illustration. JR: supervision and project administration. TT and JR: funding acquisition. All authors contributed to the article and approved the submitted version.

## Funding

JR thanks the Academy of Finland for grants 328768 and 299749. AK thanks the Finnish Cultural Foundation for grant 00220426 and Yrjö Jahnsson Foundation for grant 20217413.

## Conflict of interest

The authors declare that the research was conducted in the absence of any commercial or financial relationships that could be construed as a potential conflict of interest.

## Publisher’s note

All claims expressed in this article are solely those of the authors and do not necessarily represent those of their affiliated organizations, or those of the publisher, the editors and the reviewers. Any product that may be evaluated in this article, or claim that may be made by its manufacturer, is not guaranteed or endorsed by the publisher.
